# Supplementary material for: Targeting NLRP10 in Atopic Dermatitis: An Emerging Strategy to Modulate Epidermal Cell Death and Barrier Function
Source: Int J Mol Sci. 2025 Oct 2;26(19):9623. doi: 10.3390/ijms26199623 (PMC12525115; doi:10.3390/ijms26199623)
Supplement: Supplementary file 1 [file ijms-26-09623-s001.zip › ijms-3868179-supplementary.pdf]

GeneID	GeneName	Type	Group	Mean
ENSG00000091592.16	NLRP1	Gene	Adipose Tissue	20.9647
ENSG00000091592.16	NLRP1	Gene	Adrenal Gland	3.1995
ENSG00000091592.16	NLRP1	Gene	Bladder	12.8551
ENSG00000091592.16	NLRP1	Gene	Blood	87.2709
ENSG00000091592.16	NLRP1	Gene	Blood Vessel	12.3868
ENSG00000091592.16	NLRP1	Gene	Brain	1.6155
ENSG00000091592.16	NLRP1	Gene	Breast	17.9897
ENSG00000091592.16	NLRP1	Gene	Cervix Uteri	16.4251
ENSG00000091592.16	NLRP1	Gene	Colon	8.6692
ENSG00000091592.16	NLRP1	Gene	Esophagus	9.5975
ENSG00000091592.16	NLRP1	Gene	Fallopian Tube	11.4607
ENSG00000091592.16	NLRP1	Gene	Heart	11.8435
ENSG00000091592.16	NLRP1	Gene	Kidney	7.4378
ENSG00000091592.16	NLRP1	Gene	Liver	3.7568
ENSG00000091592.16	NLRP1	Gene	Lung	21.189
ENSG00000091592.16	NLRP1	Gene	Muscle	5.4394
ENSG00000091592.16	NLRP1	Gene	Nerve	8.5697
ENSG00000091592.16	NLRP1	Gene	Ovary	4.6372
ENSG00000091592.16	NLRP1	Gene	Pancreas	2.6943
ENSG00000091592.16	NLRP1	Gene	Pituitary	3.6328
ENSG00000091592.16	NLRP1	Gene	Prostate	10.8387
ENSG00000091592.16	NLRP1	Gene	Salivary Gland	8.5617
ENSG00000091592.16	NLRP1	Gene	Skin	28.5843
ENSG00000091592.16	NLRP1	Gene	Small Intestine	24.6612
ENSG00000091592.16	NLRP1	Gene	Spleen	41.7411
ENSG00000091592.16	NLRP1	Gene	Stomach	5.7149
ENSG00000091592.16	NLRP1	Gene	Testis	4.5411
ENSG00000091592.16	NLRP1	Gene	Thyroid	17.8256
ENSG00000091592.16	NLRP1	Gene	Uterus	13.8276
ENSG00000091592.16	NLRP1	Gene	Vagina	21.8033
ENSG00000022556.16	NLRP2	Gene	Adipose Tissue	0.2011
ENSG00000022556.16	NLRP2	Gene	Adrenal Gland	0.3133
ENSG00000022556.16	NLRP2	Gene	Bladder	1.4589
ENSG00000022556.16	NLRP2	Gene	Blood	3.8651
ENSG00000022556.16	NLRP2	Gene	Blood Vessel	0.0858
ENSG00000022556.16	NLRP2	Gene	Brain	0.8652
ENSG00000022556.16	NLRP2	Gene	Breast	1.2164
ENSG00000022556.16	NLRP2	Gene	Cervix Uteri	1.0223
ENSG00000022556.16	NLRP2	Gene	Colon	0.9696
ENSG00000022556.16	NLRP2	Gene	Esophagus	2.7818

ENSG00000022556.16	NLRP2	Gene	Fallopian Tube	1.0148
ENSG00000022556.16	NLRP2	Gene	Heart	0.0979
ENSG00000022556.16	NLRP2	Gene	Kidney	1.4161
ENSG00000022556.16	NLRP2	Gene	Liver	0.2485
ENSG00000022556.16	NLRP2	Gene	Lung	1.9007
ENSG00000022556.16	NLRP2	Gene	Muscle	0.0285
ENSG00000022556.16	NLRP2	Gene	Nerve	0.0702
ENSG00000022556.16	NLRP2	Gene	Ovary	0.2411
ENSG00000022556.16	NLRP2	Gene	Pancreas	1.8181
ENSG00000022556.16	NLRP2	Gene	Pituitary	1.8574
ENSG00000022556.16	NLRP2	Gene	Prostate	2.2432
ENSG00000022556.16	NLRP2	Gene	Salivary Gland	2.5635
ENSG00000022556.16	NLRP2	Gene	Skin	2.1218
ENSG00000022556.16	NLRP2	Gene	Small Intestine	2.2889
ENSG00000022556.16	NLRP2	Gene	Spleen	2.9188
ENSG00000022556.16	NLRP2	Gene	Stomach	2.8141
ENSG00000022556.16	NLRP2	Gene	Testis	8.8917
ENSG00000022556.16	NLRP2	Gene	Thyroid	2.6725
ENSG00000022556.16	NLRP2	Gene	Uterus	0.1185
ENSG00000022556.16	NLRP2	Gene	Vagina	4.1485
ENSG00000162711.17	NLRP3	Gene	Adipose Tissue	2.414
ENSG00000162711.17	NLRP3	Gene	Adrenal Gland	0.6337
ENSG00000162711.17	NLRP3	Gene	Bladder	0.5228
ENSG00000162711.17	NLRP3	Gene	Blood	67.3306
ENSG00000162711.17	NLRP3	Gene	Blood Vessel	1.1583
ENSG00000162711.17	NLRP3	Gene	Brain	1.1576
ENSG00000162711.17	NLRP3	Gene	Breast	0.9244
ENSG00000162711.17	NLRP3	Gene	Cervix Uteri	0.3716
ENSG00000162711.17	NLRP3	Gene	Colon	0.7229
ENSG00000162711.17	NLRP3	Gene	Esophagus	0.4333
ENSG00000162711.17	NLRP3	Gene	Fallopian Tube	1.3186
ENSG00000162711.17	NLRP3	Gene	Heart	0.6897
ENSG00000162711.17	NLRP3	Gene	Kidney	0.4412
ENSG00000162711.17	NLRP3	Gene	Liver	0.4014
ENSG00000162711.17	NLRP3	Gene	Lung	3.9568
ENSG00000162711.17	NLRP3	Gene	Muscle	0.4359
ENSG00000162711.17	NLRP3	Gene	Nerve	1.2002
ENSG00000162711.17	NLRP3	Gene	Ovary	0.3765
ENSG00000162711.17	NLRP3	Gene	Pancreas	0.3774
ENSG00000162711.17	NLRP3	Gene	Pituitary	0.5231
ENSG00000162711.17	NLRP3	Gene	Prostate	0.4869

ENSG00000162711.17	NLRP3	Gene	Salivary Gland	0.6654
ENSG00000162711.17	NLRP3	Gene	Skin	0.3094
ENSG00000162711.17	NLRP3	Gene	Small Intestine	1.1835
ENSG00000162711.17	NLRP3	Gene	Spleen	3.3611
ENSG00000162711.17	NLRP3	Gene	Stomach	0.7136
ENSG00000162711.17	NLRP3	Gene	Testis	0.2573
ENSG00000162711.17	NLRP3	Gene	Thyroid	0.4425
ENSG00000162711.17	NLRP3	Gene	Uterus	0.4649
ENSG00000162711.17	NLRP3	Gene	Vagina	0.4102
ENSG00000160505.16	NLRP4	Gene	Adipose Tissue	0.0025
ENSG00000160505.16	NLRP4	Gene	Adrenal Gland	0.0035
ENSG00000160505.16	NLRP4	Gene	Bladder	0.003
ENSG00000160505.16	NLRP4	Gene	Blood	0.0391
ENSG00000160505.16	NLRP4	Gene	Blood Vessel	0.0061
ENSG00000160505.16	NLRP4	Gene	Brain	0.0405
ENSG00000160505.16	NLRP4	Gene	Breast	0.0044
ENSG00000160505.16	NLRP4	Gene	Cervix Uteri	0.0026
ENSG00000160505.16	NLRP4	Gene	Colon	0.0037
ENSG00000160505.16	NLRP4	Gene	Esophagus	0.0027
ENSG00000160505.16	NLRP4	Gene	Fallopian Tube	0.0033
ENSG00000160505.16	NLRP4	Gene	Heart	0.0017
ENSG00000160505.16	NLRP4	Gene	Kidney	0.0024
ENSG00000160505.16	NLRP4	Gene	Liver	0.0033
ENSG00000160505.16	NLRP4	Gene	Lung	0.0049
ENSG00000160505.16	NLRP4	Gene	Muscle	0.0022
ENSG00000160505.16	NLRP4	Gene	Nerve	0.0084
ENSG00000160505.16	NLRP4	Gene	Ovary	0.0523
ENSG00000160505.16	NLRP4	Gene	Pancreas	0.0057
ENSG00000160505.16	NLRP4	Gene	Pituitary	0.0095
ENSG00000160505.16	NLRP4	Gene	Prostate	0.0082
ENSG00000160505.16	NLRP4	Gene	Salivary Gland	0.0126
ENSG00000160505.16	NLRP4	Gene	Skin	0.0046
ENSG00000160505.16	NLRP4	Gene	Small Intestine	0.0345
ENSG00000160505.16	NLRP4	Gene	Spleen	0.0396
ENSG00000160505.16	NLRP4	Gene	Stomach	0.002
ENSG00000160505.16	NLRP4	Gene	Testis	2.8621
ENSG00000160505.16	NLRP4	Gene	Thyroid	0.0132
ENSG00000160505.16	NLRP4	Gene	Uterus	0.0017
ENSG00000160505.16	NLRP4	Gene	Vagina	0.0025
ENSG00000171487.15	NLRP5	Gene	Adipose Tissue	0.0004
ENSG00000171487.15	NLRP5	Gene	Adrenal Gland	0.0006

ENSG00000171487.15	NLRP5	Gene	Bladder	0.0016
ENSG00000171487.15	NLRP5	Gene	Blood	0.0008
ENSG00000171487.15	NLRP5	Gene	Blood Vessel	0.0004
ENSG00000171487.15	NLRP5	Gene	Brain	0.0006
ENSG00000171487.15	NLRP5	Gene	Breast	0.0621
ENSG00000171487.15	NLRP5	Gene	Cervix Uteri	0.0016
ENSG00000171487.15	NLRP5	Gene	Colon	0.0004
ENSG00000171487.15	NLRP5	Gene	Esophagus	0.0007
ENSG00000171487.15	NLRP5	Gene	Fallopian Tube	0.0031
ENSG00000171487.15	NLRP5	Gene	Heart	0.0013
ENSG00000171487.15	NLRP5	Gene	Kidney	0.0003
ENSG00000171487.15	NLRP5	Gene	Liver	0.0002
ENSG00000171487.15	NLRP5	Gene	Lung	0.0003
ENSG00000171487.15	NLRP5	Gene	Muscle	0.0012
ENSG00000171487.15	NLRP5	Gene	Nerve	0.0002
ENSG00000171487.15	NLRP5	Gene	Ovary	0.3591
ENSG00000171487.15	NLRP5	Gene	Pancreas	0.0071
ENSG00000171487.15	NLRP5	Gene	Pituitary	0.0945
ENSG00000171487.15	NLRP5	Gene	Prostate	0.0075
ENSG00000171487.15	NLRP5	Gene	Salivary Gland	0.0003
ENSG00000171487.15	NLRP5	Gene	Skin	0.0004
ENSG00000171487.15	NLRP5	Gene	Small Intestine	0.0005
ENSG00000171487.15	NLRP5	Gene	Spleen	0.0004
ENSG00000171487.15	NLRP5	Gene	Stomach	0.0005
ENSG00000171487.15	NLRP5	Gene	Testis	0.1844
ENSG00000171487.15	NLRP5	Gene	Thyroid	0.0233
ENSG00000171487.15	NLRP5	Gene	Uterus	0.0005
ENSG00000171487.15	NLRP5	Gene	Vagina	0.0005
ENSG00000174885.12	NLRP6	Gene	Adipose Tissue	0.3682
ENSG00000174885.12	NLRP6	Gene	Adrenal Gland	0.0671
ENSG00000174885.12	NLRP6	Gene	Bladder	0.2663
ENSG00000174885.12	NLRP6	Gene	Blood	37.9144
ENSG00000174885.12	NLRP6	Gene	Blood Vessel	0.1857
ENSG00000174885.12	NLRP6	Gene	Brain	0.0432
ENSG00000174885.12	NLRP6	Gene	Breast	0.2183
ENSG00000174885.12	NLRP6	Gene	Cervix Uteri	0.1033
ENSG00000174885.12	NLRP6	Gene	Colon	0.3553
ENSG00000174885.12	NLRP6	Gene	Esophagus	0.0911
ENSG00000174885.12	NLRP6	Gene	Fallopian Tube	0.5206
ENSG00000174885.12	NLRP6	Gene	Heart	0.1046
ENSG00000174885.12	NLRP6	Gene	Kidney	0.9981

ENSG00000174885.12	NLRP6	Gene	Liver	1.7928
ENSG00000174885.12	NLRP6	Gene	Lung	0.7088
ENSG00000174885.12	NLRP6	Gene	Muscle	0.0416
ENSG00000174885.12	NLRP6	Gene	Nerve	0.2235
ENSG00000174885.12	NLRP6	Gene	Ovary	0.0709
ENSG00000174885.12	NLRP6	Gene	Pancreas	0.2522
ENSG00000174885.12	NLRP6	Gene	Pituitary	0.0894
ENSG00000174885.12	NLRP6	Gene	Prostate	0.1277
ENSG00000174885.12	NLRP6	Gene	Salivary Gland	0.1278
ENSG00000174885.12	NLRP6	Gene	Skin	0.0948
ENSG00000174885.12	NLRP6	Gene	Small Intestine	27.5308
ENSG00000174885.12	NLRP6	Gene	Spleen	2.5679
ENSG00000174885.12	NLRP6	Gene	Stomach	0.2777
ENSG00000174885.12	NLRP6	Gene	Testis	0.1846
ENSG00000174885.12	NLRP6	Gene	Thyroid	0.1413
ENSG00000174885.12	NLRP6	Gene	Uterus	0.0878
ENSG00000174885.12	NLRP6	Gene	Vagina	0.1184
ENSG00000167634.12	NLRP7	Gene	Adipose Tissue	0.0065
ENSG00000167634.12	NLRP7	Gene	Adrenal Gland	0.0043
ENSG00000167634.12	NLRP7	Gene	Bladder	0.0276
ENSG00000167634.12	NLRP7	Gene	Blood	0.4609
ENSG00000167634.12	NLRP7	Gene	Blood Vessel	0.0037
ENSG00000167634.12	NLRP7	Gene	Brain	0.0036
ENSG00000167634.12	NLRP7	Gene	Breast	0.0105
ENSG00000167634.12	NLRP7	Gene	Cervix Uteri	0.0109
ENSG00000167634.12	NLRP7	Gene	Colon	0.0558
ENSG00000167634.12	NLRP7	Gene	Esophagus	0.0065
ENSG00000167634.12	NLRP7	Gene	Fallopian Tube	0.0087
ENSG00000167634.12	NLRP7	Gene	Heart	0.0045
ENSG00000167634.12	NLRP7	Gene	Kidney	0.0088
ENSG00000167634.12	NLRP7	Gene	Liver	0.0227
ENSG00000167634.12	NLRP7	Gene	Lung	0.0399
ENSG00000167634.12	NLRP7	Gene	Muscle	0.0031
ENSG00000167634.12	NLRP7	Gene	Nerve	0.003
ENSG00000167634.12	NLRP7	Gene	Ovary	0.0536
ENSG00000167634.12	NLRP7	Gene	Pancreas	0.0036
ENSG00000167634.12	NLRP7	Gene	Pituitary	0.0044
ENSG00000167634.12	NLRP7	Gene	Prostate	0.0135
ENSG00000167634.12	NLRP7	Gene	Salivary Gland	0.0324
ENSG00000167634.12	NLRP7	Gene	Skin	0.0035
ENSG00000167634.12	NLRP7	Gene	Small Intestine	0.1506

ENSG00000167634.12	NLRP7	Gene	Spleen	0.4406
ENSG00000167634.12	NLRP7	Gene	Stomach	0.0346
ENSG00000167634.12	NLRP7	Gene	Testis	2.4734
ENSG00000167634.12	NLRP7	Gene	Thyroid	0.0113
ENSG00000167634.12	NLRP7	Gene	Uterus	0.0041
ENSG00000167634.12	NLRP7	Gene	Vagina	0.0229
ENSG00000179709.8	NLRP8	Gene	Adipose Tissue	0.0003
ENSG00000179709.8	NLRP8	Gene	Adrenal Gland	0.0005
ENSG00000179709.8	NLRP8	Gene	Bladder	0.0037
ENSG00000179709.8	NLRP8	Gene	Blood	0.0007
ENSG00000179709.8	NLRP8	Gene	Blood Vessel	0.0003
ENSG00000179709.8	NLRP8	Gene	Brain	0.0004
ENSG00000179709.8	NLRP8	Gene	Breast	0.0545
ENSG00000179709.8	NLRP8	Gene	Cervix Uteri	0.0029
ENSG00000179709.8	NLRP8	Gene	Colon	0.0006
ENSG00000179709.8	NLRP8	Gene	Esophagus	0.0008
ENSG00000179709.8	NLRP8	Gene	Fallopian Tube	0
ENSG00000179709.8	NLRP8	Gene	Heart	0.0007
ENSG00000179709.8	NLRP8	Gene	Kidney	0.0005
ENSG00000179709.8	NLRP8	Gene	Liver	0.0007
ENSG00000179709.8	NLRP8	Gene	Lung	0.0006
ENSG00000179709.8	NLRP8	Gene	Muscle	0.0008
ENSG00000179709.8	NLRP8	Gene	Nerve	0.0005
ENSG00000179709.8	NLRP8	Gene	Ovary	0.0042
ENSG00000179709.8	NLRP8	Gene	Pancreas	0.0006
ENSG00000179709.8	NLRP8	Gene	Pituitary	0.0002
ENSG00000179709.8	NLRP8	Gene	Prostate	0.0368
ENSG00000179709.8	NLRP8	Gene	Salivary Gland	0.0002
ENSG00000179709.8	NLRP8	Gene	Skin	0.0011
ENSG00000179709.8	NLRP8	Gene	Small Intestine	0.0003
ENSG00000179709.8	NLRP8	Gene	Spleen	0.0003
ENSG00000179709.8	NLRP8	Gene	Stomach	0.0006
ENSG00000179709.8	NLRP8	Gene	Testis	0.0496
ENSG00000179709.8	NLRP8	Gene	Thyroid	0.0003
ENSG00000179709.8	NLRP8	Gene	Uterus	0.0009
ENSG00000179709.8	NLRP8	Gene	Vagina	0.0006
ENSG00000185792.10	NLRP9	Gene	Adipose Tissue	0.0026
ENSG00000185792.10	NLRP9	Gene	Adrenal Gland	0.0027
ENSG00000185792.10	NLRP9	Gene	Bladder	0.0109
ENSG00000185792.10	NLRP9	Gene	Blood	0.003
ENSG00000185792.10	NLRP9	Gene	Blood Vessel	0.0062

ENSG00000185792.10	NLRP9	Gene	Brain	0.0034
ENSG00000185792.10	NLRP9	Gene	Breast	0.0031
ENSG00000185792.10	NLRP9	Gene	Cervix Uteri	0.0031
ENSG00000185792.10	NLRP9	Gene	Colon	0.0116
ENSG00000185792.10	NLRP9	Gene	Esophagus	0.0056
ENSG00000185792.10	NLRP9	Gene	Fallopian Tube	0.0112
ENSG00000185792.10	NLRP9	Gene	Heart	0.0019
ENSG00000185792.10	NLRP9	Gene	Kidney	0.0111
ENSG00000185792.10	NLRP9	Gene	Liver	0.0032
ENSG00000185792.10	NLRP9	Gene	Lung	0.0024
ENSG00000185792.10	NLRP9	Gene	Muscle	0.0027
ENSG00000185792.10	NLRP9	Gene	Nerve	0.0029
ENSG00000185792.10	NLRP9	Gene	Ovary	0.0999
ENSG00000185792.10	NLRP9	Gene	Pancreas	0.0367
ENSG00000185792.10	NLRP9	Gene	Pituitary	0.003
ENSG00000185792.10	NLRP9	Gene	Prostate	0.0975
ENSG00000185792.10	NLRP9	Gene	Salivary Gland	0.005
ENSG00000185792.10	NLRP9	Gene	Skin	0.0035
ENSG00000185792.10	NLRP9	Gene	Small Intestine	0.0082
ENSG00000185792.10	NLRP9	Gene	Spleen	0.0031
ENSG00000185792.10	NLRP9	Gene	Stomach	0.012
ENSG00000185792.10	NLRP9	Gene	Testis	0.101
ENSG00000185792.10	NLRP9	Gene	Thyroid	0.0016
ENSG00000185792.10	NLRP9	Gene	Uterus	0.0196
ENSG00000185792.10	NLRP9	Gene	Vagina	0.0055
ENSG00000182261.4	NLRP10	Gene	Adipose Tissue	0.0047
ENSG00000182261.4	NLRP10	Gene	Adrenal Gland	0.0017
ENSG00000182261.4	NLRP10	Gene	Bladder	0.0071
ENSG00000182261.4	NLRP10	Gene	Blood	0.004
ENSG00000182261.4	NLRP10	Gene	Blood Vessel	0.0098
ENSG00000182261.4	NLRP10	Gene	Brain	0.0023
ENSG00000182261.4	NLRP10	Gene	Breast	0.0048
ENSG00000182261.4	NLRP10	Gene	Cervix Uteri	0.0036
ENSG00000182261.4	NLRP10	Gene	Colon	0.0022
ENSG00000182261.4	NLRP10	Gene	Esophagus	0.0103
ENSG00000182261.4	NLRP10	Gene	Fallopian Tube	0.009
ENSG00000182261.4	NLRP10	Gene	Heart	0.0029
ENSG00000182261.4	NLRP10	Gene	Kidney	0.0047
ENSG00000182261.4	NLRP10	Gene	Liver	0.0028
ENSG00000182261.4	NLRP10	Gene	Lung	0.0021
ENSG00000182261.4	NLRP10	Gene	Muscle	0.004

ENSG00000182261.4	NLRP10	Gene	Nerve	0.0229
ENSG00000182261.4	NLRP10	Gene	Ovary	0.0015
ENSG00000182261.4	NLRP10	Gene	Pancreas	0.0023
ENSG00000182261.4	NLRP10	Gene	Pituitary	0.0011
ENSG00000182261.4	NLRP10	Gene	Prostate	0.0055
ENSG00000182261.4	NLRP10	Gene	Salivary Gland	0.0079
ENSG00000182261.4	NLRP10	Gene	Skin	3.6125
ENSG00000182261.4	NLRP10	Gene	Small Intestine	0.0027
ENSG00000182261.4	NLRP10	Gene	Spleen	0.0018
ENSG00000182261.4	NLRP10	Gene	Stomach	0.0041
ENSG00000182261.4	NLRP10	Gene	Testis	0.03
ENSG00000182261.4	NLRP10	Gene	Thyroid	0.0015
ENSG00000182261.4	NLRP10	Gene	Uterus	0.0011
ENSG00000182261.4	NLRP10	Gene	Vagina	0.0404
ENSG00000179873.14	NLRP11	Gene	Adipose Tissue	0.0052
ENSG00000179873.14	NLRP11	Gene	Adrenal Gland	0.022
ENSG00000179873.14	NLRP11	Gene	Bladder	0.0083
ENSG00000179873.14	NLRP11	Gene	Blood	0.0175
ENSG00000179873.14	NLRP11	Gene	Blood Vessel	0.0022
ENSG00000179873.14	NLRP11	Gene	Brain	0.0074
ENSG00000179873.14	NLRP11	Gene	Breast	0.0063
ENSG00000179873.14	NLRP11	Gene	Cervix Uteri	0.0126
ENSG00000179873.14	NLRP11	Gene	Colon	0.02
ENSG00000179873.14	NLRP11	Gene	Esophagus	0.0038
ENSG00000179873.14	NLRP11	Gene	Fallopian Tube	0.0352
ENSG00000179873.14	NLRP11	Gene	Heart	0.0023
ENSG00000179873.14	NLRP11	Gene	Kidney	0.0755
ENSG00000179873.14	NLRP11	Gene	Liver	0.4261
ENSG00000179873.14	NLRP11	Gene	Lung	0.0072
ENSG00000179873.14	NLRP11	Gene	Muscle	0.0023
ENSG00000179873.14	NLRP11	Gene	Nerve	0.0064
ENSG00000179873.14	NLRP11	Gene	Ovary	0.1832
ENSG00000179873.14	NLRP11	Gene	Pancreas	0.0999
ENSG00000179873.14	NLRP11	Gene	Pituitary	0.0267
ENSG00000179873.14	NLRP11	Gene	Prostate	0.0105
ENSG00000179873.14	NLRP11	Gene	Salivary Gland	0.0258
ENSG00000179873.14	NLRP11	Gene	Skin	0.0045
ENSG00000179873.14	NLRP11	Gene	Small Intestine	0.0516
ENSG00000179873.14	NLRP11	Gene	Spleen	0.0616
ENSG00000179873.14	NLRP11	Gene	Stomach	0.0784
ENSG00000179873.14	NLRP11	Gene	Testis	0.9249

ENSG00000179873.14	NLRP11	Gene	Thyroid	0.0105
ENSG00000179873.14	NLRP11	Gene	Uterus	0.0105
ENSG00000179873.14	NLRP11	Gene	Vagina	0.0048
ENSG00000142405.21	NLRP12	Gene	Adipose Tissue	0.3034
ENSG00000142405.21	NLRP12	Gene	Adrenal Gland	0.0725
ENSG00000142405.21	NLRP12	Gene	Bladder	0.1062
ENSG00000142405.21	NLRP12	Gene	Blood	70.4552
ENSG00000142405.21	NLRP12	Gene	Blood Vessel	0.1448
ENSG00000142405.21	NLRP12	Gene	Brain	0.0484
ENSG00000142405.21	NLRP12	Gene	Breast	0.1131
ENSG00000142405.21	NLRP12	Gene	Cervix Uteri	0.0694
ENSG00000142405.21	NLRP12	Gene	Colon	0.0773
ENSG00000142405.21	NLRP12	Gene	Esophagus	0.077
ENSG00000142405.21	NLRP12	Gene	Fallopian Tube	0.3237
ENSG00000142405.21	NLRP12	Gene	Heart	0.1767
ENSG00000142405.21	NLRP12	Gene	Kidney	0.0799
ENSG00000142405.21	NLRP12	Gene	Liver	0.1519
ENSG00000142405.21	NLRP12	Gene	Lung	1.0186
ENSG00000142405.21	NLRP12	Gene	Muscle	0.0672
ENSG00000142405.21	NLRP12	Gene	Nerve	0.1261
ENSG00000142405.21	NLRP12	Gene	Ovary	0.0486
ENSG00000142405.21	NLRP12	Gene	Pancreas	0.0981
ENSG00000142405.21	NLRP12	Gene	Pituitary	0.0527
ENSG00000142405.21	NLRP12	Gene	Prostate	0.0716
ENSG00000142405.21	NLRP12	Gene	Salivary Gland	0.1849
ENSG00000142405.21	NLRP12	Gene	Skin	0.0333
ENSG00000142405.21	NLRP12	Gene	Small Intestine	0.0837
ENSG00000142405.21	NLRP12	Gene	Spleen	3.2361
ENSG00000142405.21	NLRP12	Gene	Stomach	0.1037
ENSG00000142405.21	NLRP12	Gene	Testis	0.1252
ENSG00000142405.21	NLRP12	Gene	Thyroid	0.066
ENSG00000142405.21	NLRP12	Gene	Uterus	0.074
ENSG00000142405.21	NLRP12	Gene	Vagina	0.0705
ENSG00000173572.11	NLRP13	Gene	Adipose Tissue	0.0002
ENSG00000173572.11	NLRP13	Gene	Adrenal Gland	0.0003
ENSG00000173572.11	NLRP13	Gene	Bladder	0.0074
ENSG00000173572.11	NLRP13	Gene	Blood	0.0017
ENSG00000173572.11	NLRP13	Gene	Blood Vessel	0.0004
ENSG00000173572.11	NLRP13	Gene	Brain	0.0006
ENSG00000173572.11	NLRP13	Gene	Breast	0.3273
ENSG00000173572.11	NLRP13	Gene	Cervix Uteri	0.0021

ENSG00000173572.11	NLRP13	Gene	Colon	0.0004
ENSG00000173572.11	NLRP13	Gene	Esophagus	0.0007
ENSG00000173572.11	NLRP13	Gene	Fallopian Tube	0
ENSG00000173572.11	NLRP13	Gene	Heart	0.0007
ENSG00000173572.11	NLRP13	Gene	Kidney	0.0003
ENSG00000173572.11	NLRP13	Gene	Liver	0.0005
ENSG00000173572.11	NLRP13	Gene	Lung	0.0011
ENSG00000173572.11	NLRP13	Gene	Muscle	0.0011
ENSG00000173572.11	NLRP13	Gene	Nerve	0.0004
ENSG00000173572.11	NLRP13	Gene	Ovary	0.0741
ENSG00000173572.11	NLRP13	Gene	Pancreas	0.0009
ENSG00000173572.11	NLRP13	Gene	Pituitary	0.0005
ENSG00000173572.11	NLRP13	Gene	Prostate	0.0856
ENSG00000173572.11	NLRP13	Gene	Salivary Gland	0.0007
ENSG00000173572.11	NLRP13	Gene	Skin	0.0047
ENSG00000173572.11	NLRP13	Gene	Small Intestine	0.0004
ENSG00000173572.11	NLRP13	Gene	Spleen	0.0006
ENSG00000173572.11	NLRP13	Gene	Stomach	0.0005
ENSG00000173572.11	NLRP13	Gene	Testis	0.0639
ENSG00000173572.11	NLRP13	Gene	Thyroid	0.0004
ENSG00000173572.11	NLRP13	Gene	Uterus	0.0006
ENSG00000173572.11	NLRP13	Gene	Vagina	0.0004
ENSG00000158077.4	NLRP14	Gene	Adipose Tissue	0.0104
ENSG00000158077.4	NLRP14	Gene	Adrenal Gland	0.0115
ENSG00000158077.4	NLRP14	Gene	Bladder	0.0214
ENSG00000158077.4	NLRP14	Gene	Blood	0.009
ENSG00000158077.4	NLRP14	Gene	Blood Vessel	0.0122
ENSG00000158077.4	NLRP14	Gene	Brain	0.0506
ENSG00000158077.4	NLRP14	Gene	Breast	0.0191
ENSG00000158077.4	NLRP14	Gene	Cervix Uteri	0.024
ENSG00000158077.4	NLRP14	Gene	Colon	0.0233
ENSG00000158077.4	NLRP14	Gene	Esophagus	0.0088
ENSG00000158077.4	NLRP14	Gene	Fallopian Tube	0.0451
ENSG00000158077.4	NLRP14	Gene	Heart	0.0152
ENSG00000158077.4	NLRP14	Gene	Kidney	0.0627
ENSG00000158077.4	NLRP14	Gene	Liver	0.0718
ENSG00000158077.4	NLRP14	Gene	Lung	0.046
ENSG00000158077.4	NLRP14	Gene	Muscle	0.0045
ENSG00000158077.4	NLRP14	Gene	Nerve	0.0894
ENSG00000158077.4	NLRP14	Gene	Ovary	0.068
ENSG00000158077.4	NLRP14	Gene	Pancreas	0.0185

ENSG00000158077.4	NLRP14	Gene	Pituitary	0.0265
ENSG00000158077.4	NLRP14	Gene	Prostate	0.0258
ENSG00000158077.4	NLRP14	Gene	Salivary Gland	0.047
ENSG00000158077.4	NLRP14	Gene	Skin	0.0109
ENSG00000158077.4	NLRP14	Gene	Small Intestine	0.0391
ENSG00000158077.4	NLRP14	Gene	Spleen	0.0468
ENSG00000158077.4	NLRP14	Gene	Stomach	0.0149
ENSG00000158077.4	NLRP14	Gene	Testis	0.3906
ENSG00000158077.4	NLRP14	Gene	Thyroid	0.0388
ENSG00000158077.4	NLRP14	Gene	Uterus	0.0191
ENSG00000158077.4	NLRP14	Gene	Vagina	0.0226
ENSG00000179583.19	CIITA	Gene	Adipose Tissue	5.0255
ENSG00000179583.19	CIITA	Gene	Adrenal Gland	4.0176
ENSG00000179583.19	CIITA	Gene	Bladder	2.4246
ENSG00000179583.19	CIITA	Gene	Blood	10.7649
ENSG00000179583.19	CIITA	Gene	Blood Vessel	2.6379
ENSG00000179583.19	CIITA	Gene	Brain	1.1525
ENSG00000179583.19	CIITA	Gene	Breast	4.7885
ENSG00000179583.19	CIITA	Gene	Cervix Uteri	2.9798
ENSG00000179583.19	CIITA	Gene	Colon	2.3179
ENSG00000179583.19	CIITA	Gene	Esophagus	2.4901
ENSG00000179583.19	CIITA	Gene	Fallopian Tube	3.3963
ENSG00000179583.19	CIITA	Gene	Heart	2.6803
ENSG00000179583.19	CIITA	Gene	Kidney	4.9499
ENSG00000179583.19	CIITA	Gene	Liver	2.1368
ENSG00000179583.19	CIITA	Gene	Lung	9.8807
ENSG00000179583.19	CIITA	Gene	Muscle	1.5597
ENSG00000179583.19	CIITA	Gene	Nerve	6.5591
ENSG00000179583.19	CIITA	Gene	Ovary	1.2964
ENSG00000179583.19	CIITA	Gene	Pancreas	1.7282
ENSG00000179583.19	CIITA	Gene	Pituitary	2.1067
ENSG00000179583.19	CIITA	Gene	Prostate	3.2258
ENSG00000179583.19	CIITA	Gene	Salivary Gland	3.1552
ENSG00000179583.19	CIITA	Gene	Skin	2.6328
ENSG00000179583.19	CIITA	Gene	Small Intestine	12.2281
ENSG00000179583.19	CIITA	Gene	Spleen	16.4192
ENSG00000179583.19	CIITA	Gene	Stomach	2.8804
ENSG00000179583.19	CIITA	Gene	Testis	0.563
ENSG00000179583.19	CIITA	Gene	Thyroid	3.3273
ENSG00000179583.19	CIITA	Gene	Uterus	2.8249
ENSG00000179583.19	CIITA	Gene	Vagina	2.6514

ENSG00000249437.7	NAIP	Gene	Adipose Tissue	6.6502
ENSG00000249437.7	NAIP	Gene	Adrenal Gland	5.0398
ENSG00000249437.7	NAIP	Gene	Bladder	5.3674
ENSG00000249437.7	NAIP	Gene	Blood	164.8595
ENSG00000249437.7	NAIP	Gene	Blood Vessel	4.7652
ENSG00000249437.7	NAIP	Gene	Brain	6.1485
ENSG00000249437.7	NAIP	Gene	Breast	6.1104
ENSG00000249437.7	NAIP	Gene	Cervix Uteri	8.8462
ENSG00000249437.7	NAIP	Gene	Colon	5.4263
ENSG00000249437.7	NAIP	Gene	Esophagus	4.1116
ENSG00000249437.7	NAIP	Gene	Fallopian Tube	8.4409
ENSG00000249437.7	NAIP	Gene	Heart	4.6157
ENSG00000249437.7	NAIP	Gene	Kidney	5.0629
ENSG00000249437.7	NAIP	Gene	Liver	5.8656
ENSG00000249437.7	NAIP	Gene	Lung	10.3715
ENSG00000249437.7	NAIP	Gene	Muscle	1.5826
ENSG00000249437.7	NAIP	Gene	Nerve	7.5301
ENSG00000249437.7	NAIP	Gene	Ovary	7.0016
ENSG00000249437.7	NAIP	Gene	Pancreas	5.6492
ENSG00000249437.7	NAIP	Gene	Pituitary	8.2944
ENSG00000249437.7	NAIP	Gene	Prostate	7.4506
ENSG00000249437.7	NAIP	Gene	Salivary Gland	4.1953
ENSG00000249437.7	NAIP	Gene	Skin	4.5645
ENSG00000249437.7	NAIP	Gene	Small Intestine	7.3638
ENSG00000249437.7	NAIP	Gene	Spleen	34.4925
ENSG00000249437.7	NAIP	Gene	Stomach	4.5507
ENSG00000249437.7	NAIP	Gene	Testis	8.4676
ENSG00000249437.7	NAIP	Gene	Thyroid	9.926
ENSG00000249437.7	NAIP	Gene	Uterus	6.6394
ENSG00000249437.7	NAIP	Gene	Vagina	6.6898
ENSG00000091106.19	NLRC4	Gene	Adipose Tissue	1.0005
ENSG00000091106.19	NLRC4	Gene	Adrenal Gland	0.9678
ENSG00000091106.19	NLRC4	Gene	Bladder	0.4466
ENSG00000091106.19	NLRC4	Gene	Blood	68.7727
ENSG00000091106.19	NLRC4	Gene	Blood Vessel	0.48
ENSG00000091106.19	NLRC4	Gene	Brain	0.4713
ENSG00000091106.19	NLRC4	Gene	Breast	0.5063
ENSG00000091106.19	NLRC4	Gene	Cervix Uteri	0.2775
ENSG00000091106.19	NLRC4	Gene	Colon	0.4234
ENSG00000091106.19	NLRC4	Gene	Esophagus	0.308
ENSG00000091106.19	NLRC4	Gene	Fallopian Tube	0.6226

ENSG00000091106.19	NLRC4	Gene	Heart	0.8039
ENSG00000091106.19	NLRC4	Gene	Kidney	0.373
ENSG00000091106.19	NLRC4	Gene	Liver	0.7162
ENSG00000091106.19	NLRC4	Gene	Lung	2.2981
ENSG00000091106.19	NLRC4	Gene	Muscle	0.6736
ENSG00000091106.19	NLRC4	Gene	Nerve	0.3724
ENSG00000091106.19	NLRC4	Gene	Ovary	0.2707
ENSG00000091106.19	NLRC4	Gene	Pancreas	0.5348
ENSG00000091106.19	NLRC4	Gene	Pituitary	0.493
ENSG00000091106.19	NLRC4	Gene	Prostate	0.3299
ENSG00000091106.19	NLRC4	Gene	Salivary Gland	0.3521
ENSG00000091106.19	NLRC4	Gene	Skin	0.1514
ENSG00000091106.19	NLRC4	Gene	Small Intestine	0.7235
ENSG00000091106.19	NLRC4	Gene	Spleen	7.2733
ENSG00000091106.19	NLRC4	Gene	Stomach	0.4145
ENSG00000091106.19	NLRC4	Gene	Testis	0.3789
ENSG00000091106.19	NLRC4	Gene	Thyroid	0.2851
ENSG00000091106.19	NLRC4	Gene	Uterus	0.289
ENSG00000091106.19	NLRC4	Gene	Vagina	0.2736
ENSG00000106100.11	NOD1	Gene	Adipose Tissue	9.2929
ENSG00000106100.11	NOD1	Gene	Adrenal Gland	2.2015
ENSG00000106100.11	NOD1	Gene	Bladder	6.4259
ENSG00000106100.11	NOD1	Gene	Blood	3.6766
ENSG00000106100.11	NOD1	Gene	Blood Vessel	4.4433
ENSG00000106100.11	NOD1	Gene	Brain	1.2402
ENSG00000106100.11	NOD1	Gene	Breast	8.6808
ENSG00000106100.11	NOD1	Gene	Cervix Uteri	4.6271
ENSG00000106100.11	NOD1	Gene	Colon	4.4232
ENSG00000106100.11	NOD1	Gene	Esophagus	5.573
ENSG00000106100.11	NOD1	Gene	Fallopian Tube	6.1793
ENSG00000106100.11	NOD1	Gene	Heart	6.9896
ENSG00000106100.11	NOD1	Gene	Kidney	3.8147
ENSG00000106100.11	NOD1	Gene	Liver	1.606
ENSG00000106100.11	NOD1	Gene	Lung	9.1885
ENSG00000106100.11	NOD1	Gene	Muscle	2.5185
ENSG00000106100.11	NOD1	Gene	Nerve	8.1762
ENSG00000106100.11	NOD1	Gene	Ovary	9.6823
ENSG00000106100.11	NOD1	Gene	Pancreas	1.8573
ENSG00000106100.11	NOD1	Gene	Pituitary	2.9904
ENSG00000106100.11	NOD1	Gene	Prostate	3.9052
ENSG00000106100.11	NOD1	Gene	Salivary Gland	4.7733

ENSG00000106100.11	NOD1	Gene	Skin	4.7189
ENSG00000106100.11	NOD1	Gene	Small Intestine	5.0181
ENSG00000106100.11	NOD1	Gene	Spleen	8.5353
ENSG00000106100.11	NOD1	Gene	Stomach	4.2754
ENSG00000106100.11	NOD1	Gene	Testis	1.3182
ENSG00000106100.11	NOD1	Gene	Thyroid	10.656
ENSG00000106100.11	NOD1	Gene	Uterus	6.0075
ENSG00000106100.11	NOD1	Gene	Vagina	4.3786
ENSG00000167207.13	NOD2	Gene	Adipose Tissue	0.9471
ENSG00000167207.13	NOD2	Gene	Adrenal Gland	0.4041
ENSG00000167207.13	NOD2	Gene	Bladder	0.6769
ENSG00000167207.13	NOD2	Gene	Blood	28.9358
ENSG00000167207.13	NOD2	Gene	Blood Vessel	1.2092
ENSG00000167207.13	NOD2	Gene	Brain	0.2723
ENSG00000167207.13	NOD2	Gene	Breast	0.6874
ENSG00000167207.13	NOD2	Gene	Cervix Uteri	2.322
ENSG00000167207.13	NOD2	Gene	Colon	0.6652
ENSG00000167207.13	NOD2	Gene	Esophagus	3.8047
ENSG00000167207.13	NOD2	Gene	Fallopian Tube	0.7428
ENSG00000167207.13	NOD2	Gene	Heart	0.5983
ENSG00000167207.13	NOD2	Gene	Kidney	0.4698
ENSG00000167207.13	NOD2	Gene	Liver	1.208
ENSG00000167207.13	NOD2	Gene	Lung	3.3553
ENSG00000167207.13	NOD2	Gene	Muscle	0.4875
ENSG00000167207.13	NOD2	Gene	Nerve	1.1023
ENSG00000167207.13	NOD2	Gene	Ovary	0.3676
ENSG00000167207.13	NOD2	Gene	Pancreas	0.2784
ENSG00000167207.13	NOD2	Gene	Pituitary	0.3438
ENSG00000167207.13	NOD2	Gene	Prostate	0.7316
ENSG00000167207.13	NOD2	Gene	Salivary Gland	3.4777
ENSG00000167207.13	NOD2	Gene	Skin	8.7148
ENSG00000167207.13	NOD2	Gene	Small Intestine	1.056
ENSG00000167207.13	NOD2	Gene	Spleen	3.2494
ENSG00000167207.13	NOD2	Gene	Stomach	0.463
ENSG00000167207.13	NOD2	Gene	Testis	0.3891
ENSG00000167207.13	NOD2	Gene	Thyroid	0.3881
ENSG00000167207.13	NOD2	Gene	Uterus	0.4402
ENSG00000167207.13	NOD2	Gene	Vagina	9.2907
ENSG00000167984.18	NLRC3	Gene	Adipose Tissue	0.7428
ENSG00000167984.18	NLRC3	Gene	Adrenal Gland	0.5456
ENSG00000167984.18	NLRC3	Gene	Bladder	0.9088

ENSG00000167984.18	NLRC3	Gene	Blood	8.0359
ENSG00000167984.18	NLRC3	Gene	Blood Vessel	0.6314
ENSG00000167984.18	NLRC3	Gene	Brain	0.946
ENSG00000167984.18	NLRC3	Gene	Breast	0.5963
ENSG00000167984.18	NLRC3	Gene	Cervix Uteri	0.7608
ENSG00000167984.18	NLRC3	Gene	Colon	1.0629
ENSG00000167984.18	NLRC3	Gene	Esophagus	0.6883
ENSG00000167984.18	NLRC3	Gene	Fallopian Tube	1.0348
ENSG00000167984.18	NLRC3	Gene	Heart	0.8767
ENSG00000167984.18	NLRC3	Gene	Kidney	1.112
ENSG00000167984.18	NLRC3	Gene	Liver	0.8454
ENSG00000167984.18	NLRC3	Gene	Lung	2.0436
ENSG00000167984.18	NLRC3	Gene	Muscle	0.5258
ENSG00000167984.18	NLRC3	Gene	Nerve	0.5801
ENSG00000167984.18	NLRC3	Gene	Ovary	0.598
ENSG00000167984.18	NLRC3	Gene	Pancreas	0.4934
ENSG00000167984.18	NLRC3	Gene	Pituitary	0.7785
ENSG00000167984.18	NLRC3	Gene	Prostate	0.9596
ENSG00000167984.18	NLRC3	Gene	Salivary Gland	0.7369
ENSG00000167984.18	NLRC3	Gene	Skin	0.3573
ENSG00000167984.18	NLRC3	Gene	Small Intestine	5.6213
ENSG00000167984.18	NLRC3	Gene	Spleen	9.4248
ENSG00000167984.18	NLRC3	Gene	Stomach	1.0669
ENSG00000167984.18	NLRC3	Gene	Testis	0.8535
ENSG00000167984.18	NLRC3	Gene	Thyroid	1.3581
ENSG00000167984.18	NLRC3	Gene	Uterus	0.7253
ENSG00000167984.18	NLRC3	Gene	Vagina	0.7394
ENSG00000140853.15	NLRC5	Gene	Adipose Tissue	7.3452
ENSG00000140853.15	NLRC5	Gene	Adrenal Gland	5.3539
ENSG00000140853.15	NLRC5	Gene	Bladder	4.6478
ENSG00000140853.15	NLRC5	Gene	Blood	64.7804
ENSG00000140853.15	NLRC5	Gene	Blood Vessel	3.4258
ENSG00000140853.15	NLRC5	Gene	Brain	1.5881
ENSG00000140853.15	NLRC5	Gene	Breast	5.9171
ENSG00000140853.15	NLRC5	Gene	Cervix Uteri	4.6763
ENSG00000140853.15	NLRC5	Gene	Colon	6.7966
ENSG00000140853.15	NLRC5	Gene	Esophagus	4.1372
ENSG00000140853.15	NLRC5	Gene	Fallopian Tube	4.5568
ENSG00000140853.15	NLRC5	Gene	Heart	3.8846
ENSG00000140853.15	NLRC5	Gene	Kidney	4.8015
ENSG00000140853.15	NLRC5	Gene	Liver	4.97

ENSG00000140853.15	NLRC5	Gene	Lung	11.942
ENSG00000140853.15	NLRC5	Gene	Muscle	1.9375
ENSG00000140853.15	NLRC5	Gene	Nerve	3.4688
ENSG00000140853.15	NLRC5	Gene	Ovary	3.0335
ENSG00000140853.15	NLRC5	Gene	Pancreas	2.673
ENSG00000140853.15	NLRC5	Gene	Pituitary	10.2338
ENSG00000140853.15	NLRC5	Gene	Prostate	4.8218
ENSG00000140853.15	NLRC5	Gene	Salivary Gland	3.6891
ENSG00000140853.15	NLRC5	Gene	Skin	9.624
ENSG00000140853.15	NLRC5	Gene	Small Intestine	17.4874
ENSG00000140853.15	NLRC5	Gene	Spleen	35
ENSG00000140853.15	NLRC5	Gene	Stomach	4.7859
ENSG00000140853.15	NLRC5	Gene	Testis	0.7368
ENSG00000140853.15	NLRC5	Gene	Thyroid	5.3175
ENSG00000140853.15	NLRC5	Gene	Uterus	6.926
ENSG00000140853.15	NLRC5	Gene	Vagina	5.525
ENSG00000160703.16	NLRX1	Gene	Adipose Tissue	5.386
ENSG00000160703.16	NLRX1	Gene	Adrenal Gland	8.2847
ENSG00000160703.16	NLRX1	Gene	Bladder	7.403
ENSG00000160703.16	NLRX1	Gene	Blood	17.5138
ENSG00000160703.16	NLRX1	Gene	Blood Vessel	5.9823
ENSG00000160703.16	NLRX1	Gene	Brain	3.1711
ENSG00000160703.16	NLRX1	Gene	Breast	5.4061
ENSG00000160703.16	NLRX1	Gene	Cervix Uteri	6.1144
ENSG00000160703.16	NLRX1	Gene	Colon	7.1298
ENSG00000160703.16	NLRX1	Gene	Esophagus	17.3714
ENSG00000160703.16	NLRX1	Gene	Fallopian Tube	6.4288
ENSG00000160703.16	NLRX1	Gene	Heart	10.497
ENSG00000160703.16	NLRX1	Gene	Kidney	6.0838
ENSG00000160703.16	NLRX1	Gene	Liver	6.2258
ENSG00000160703.16	NLRX1	Gene	Lung	4.1504
ENSG00000160703.16	NLRX1	Gene	Muscle	14.1074
ENSG00000160703.16	NLRX1	Gene	Nerve	5.083
ENSG00000160703.16	NLRX1	Gene	Ovary	3.0879
ENSG00000160703.16	NLRX1	Gene	Pancreas	6.4754
ENSG00000160703.16	NLRX1	Gene	Pituitary	3.894
ENSG00000160703.16	NLRX1	Gene	Prostate	6.5132
ENSG00000160703.16	NLRX1	Gene	Salivary Gland	7.8238
ENSG00000160703.16	NLRX1	Gene	Skin	12.9995
ENSG00000160703.16	NLRX1	Gene	Small Intestine	4.9022
ENSG00000160703.16	NLRX1	Gene	Spleen	5.8372

ENSG00000160703.16	NLRX1	Gene	Stomach	5.6931
ENSG00000160703.16	NLRX1	Gene	Testis	2.6374
ENSG00000160703.16	NLRX1	Gene	Thyroid	6.0859
ENSG00000160703.16	NLRX1	Gene	Uterus	6.5288
ENSG00000160703.16	NLRX1	Gene	Vagina	12.021
